# Untargeted Metabolomics Reveals New Markers of Food Processing for Strawberry and Apple Purees

**DOI:** 10.3390/molecules27217275

**Published:** 2022-10-26

**Authors:** Gabriela Salazar-Orbea, Rocío García-Villalba, Luis M. Sánchez-Siles, Francisco A. Tomás-Barberán, Carlos J. García

**Affiliations:** 1 Quality, Safety and Bioactivity of Plant-Derived Foods, Centro de Edafología y Biología Aplicada del Segura-Consejo Superior de Investigaciones Científicas (CEBAS-CSIC), 30100 Murcia, Spain; 2 Research and Nutrition Department, Hero Group, 30820 Alcantarilla, Spain; 3 Institute for Research and Nutrition, Hero Group, 5600 Lenzburg, Switzerland

**Keywords:** untargeted metabolomics, markers, food processing, processing degree

## Abstract

In general, food processing and its conditions affect nutrients, bioactive compounds, and sensory characteristics of food products. This research aims to use a non-targeted metabolomics approach based on UPLC-ESI-QTOF-MS to determine how fruit processing can affect the metabolic profile of fruits and, through a comprehensive metabolic analysis, identify possible markers to assess their degree of processing. The present study uses a real case from the food industry to evaluate markers of the processing of strawberry and apple purees industrially elaborated with different processing techniques and conditions. The results from the multivariate analysis revealed that samples were grouped according to the type of processing, evidencing changes in their metabolic profiles and an apparent temperature-dependent effect. These metabolic profiles showed changes according to the relevance of thermal conditions but also according to the exclusively cold treatment, in the case of strawberry puree, and the pressure treatment, in the case of apple puree. After data analysis, seven metabolites were identified and proposed as processing markers: pyroglutamic acid, pteroyl-D-glutamic acid, 2-hydroxy-5-methoxy benzoic acid, and 2-hydroxybenzoic acid β-d-glucoside in strawberry and di-hydroxycinnamic acid glucuronide, caffeic acid and lysoPE(18:3(9Z,12Z,15Z)/0:0) in apple purees. The use of these markers may potentially help to objectively measure the degree of food processing and help to clarify the controversial narrative on ultra-processed foods.

## 1. Introduction

Food processing refers to different unit operations that modify foodstuff chemically and physically to extend its shelf life and ensure food quality and safety [1]. Some traditional unit operations used in the fruit industry include peeling, chopping, crushing, sieving, and thermal treatments [2]. Many studies have determined how different processing techniques and conditions affect some sensitive nutrients (e.g., vitamins) and bioactive compounds, as well as the quality characteristics of fruit products [3,4].

Industries must ensure the supply of fresh fruits to manufacture fruit products throughout the year. However, sometimes fruits are processed in advance to be used as raw materials due to the difficulty of local production or fruits (fresh) not available in all seasons. Overall, thermal treatment is the most used processing technology to produce fruit products such as purees, jams, juices, concentrates, and baby foods [1]. Nevertheless, in light of an increasing demand of consumers towards minimally processed and healthier products [5], non-thermal technologies such as high-pressure processing (HPP), cold atmospheric plasma, and pulsed electric fields, among others, have been developed [6]. It has been shown that the degree of processing in thermal and non-thermal processing techniques affects the stability of polyphenols in fruit products [3]. However, the degree of processing has not been fully assessed yet, as many authors categorize the degree of processing according to the number of ingredients in a food product or to the extent and purpose of food processing, and not based on the process itself [7,8,9]. 

Therefore, defining the degree of processing of the fruit products will help assess the preservation level of the phenolic compounds and the quality characteristics of the final product (e.g., fruit juices, fruit purees, jams). This represents a significant challenge for industries and consumers. A potential strategy would be to study the overall metabolic profile of the final product, which could be affected by the degree of processing. Metabolomics-based approaches have been demonstrated to be a powerful tool for identifying changes in food metabolic profiles due to processing [10]. In particular, untargeted metabolomics has been used to study modifications after minimal processing and storage of meat, fruit, and vegetable products [11,12,13]. A previous study examining how processing (thermal pasteurization, pulsed electric field, and high-pressure processing) influences the quality attributes of cloudy apple juice using a targeted and non-targeted metabolomics approach revealed that heat treatment resulted in brighter color together with increased stability of cloudiness in the juice, mainly as a result of inactivation of polyphenol oxidase, peroxidase and pectin methylesterase. However, this heat treatment reduced most of the volatile compounds, principally esters, and induced the formation of off-odor compounds [14]. In addition, Utpott et al.’s [10] review recently reported changes in the metabolic profile due to thermal treatments, drying technologies, fermentation, and chemical and enzymatic treatments. However, the elucidation of potential markers is challenging [15]. Therefore, there is a need for research that examines the influence of thermal and non-thermal processing techniques on the metabolites of fruit products to determine common processing markers to provide helpful information to consumers, industries, and governmental food regulators.

This study will focus on strawberries and apples, two of the most used fruits in the food industry, not only because of their pleasant flavor and sensory characteristics but also due to the presence of bioactive compounds to which their potential health benefits are attributed [16]. The aim is to determine potential markers of food processing in strawberries and apples subjected to different industrial processing techniques, as previously described [17], to produce purees using an untargeted metabolomics approach.

## 2. Results

### 2.1. Multivariate Model Analysis

#### 2.1.1. Multivariate Model Analysis of Strawberry

**Figure 1:**
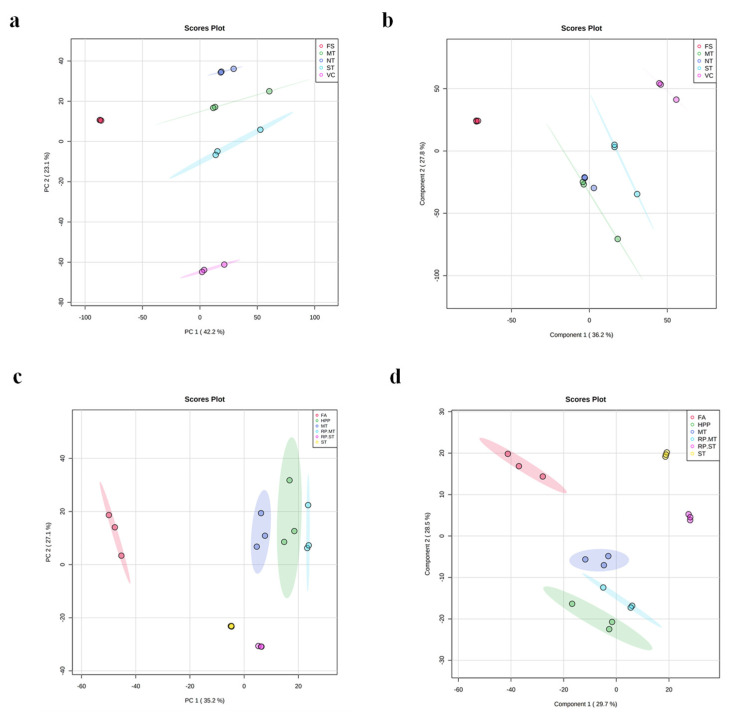
PCA and PLS-DA model plots. Strawberry: (**a**) PCA plot, (**b**) PLS-DA plot. Apple: (**c**) PCA plot, (**d**) PLS-DA plot. Strawberry samples: Fresh strawberries (FS), No heat treatment (NT), mild heat treatment (MT), standard thermal treatment (ST), vacuum concentration (VC). Apple samples: Fresh apple (FA), high-pressure processing (HPP), mild heat treatment (MT), standard thermal treatment (ST), reprocessed mild heat treatment (RP.MT), reprocessed standard thermal treatment (RP.ST).

The pre-processing operations gave a data matrix from the full dataset based on 4554 entities (ions that present the necessary features to be a metabolite) for negative mode. In the PCA analysis, the first two principal components (PC1 and PC2) explained 65.3% of the total variability in the negative mode (Figure 1a). Samples were grouped according to the processing technique (Fresh strawberries (FS), No heat treatment (NT), mild heat treatment (MT), standard thermal treatment, vacuum concentration (VC)), evidencing changes in their metabolic profiles. The calculated PLS-DA model, based on 15 samples, described 96.8% of the variance (R^2^ = 0.968) according to the cross-validation prediction of Q2 = 0.612 (Figure 1b) in negative polarity. The discrimination models showed significant differences between FS and VC, but not very high discrimination across the cold-crushing processing samples NT and MT, which showed similar metabolic profiles (Figure 1b). The summarized variance explained by component 1 classified the variables’ thermal treatment effect, whereas component 2 classified the variables’ cold crushing effect. These results showed how the temperature variation affected the discrimination of samples. Samples were classified along component 1 according to the relevance of the thermal treatment (FS-NT/MT-ST-VC). On the other hand, the samples subjected to cold crushing processing (NT and MT) were classified together in the model and separated by component 2 from fresh samples (FS) and the hot crushing processing samples (ST and VC). The results show the sample metabolome variability according to the temperature used during the processing stages.

#### 2.1.2. Multivariate Model Analysis of Apple

For negative mode, the pre-processing procedures gave a data matrix from the full dataset based on 2551. PC1 and PC2 explained 62.3% of the variance in the negative mode (Figure 1c). The global trend of the data variation in the PCA plots describes a clear sample grouping affected by processing treatment [Fresh apple (FA), High-pressure processing (HPP), mild heat treatment (MT), standard thermal treatment (ST), reprocessed mild heat treatment (RP.MT), reprocessed standard thermal treatment (RP.ST), with no significant outliers. The PCA model showed accurate technical reproducibility. The calculated PLS-DA model based on 18 samples described 98.8% of the variation (R^2^ = 0.988) according to the cross-validation prediction of Q2 = 0.911 for negative polarity (Figure 1d). The summarized variance explained by component 1 exclusively classified the variables’ thermal treatment effect, whereas that component 2 classified the variables’ cold crushing effect. These results showed how the cold-crushing treatment could differentiate the metabolome of these samples, similar to HPP, MT, and RP.MT treatments.

In summary, the variance explained by component 1 discriminated the fresh product, in both study cases, from the treatments mainly affected by the temperature observed in apple puree, while component 2 grouped the metabolomes affected by cold crushing.

### 2.2. Metabolites Trend and Markers Identification

After evaluating the multivariate model and the application of univariate operations, a list of candidate markers was obtained.

Strawberry. The filtering and statistics layers resulted in 2902 entities in the negative mode. According to the highest VIP value score (VIP > 1), the accuracy of the tentative database identification, and the p-value, a final list of 1444 entities was obtained. Finally, four entities were identified using accurate mass and MS/MS fragmentation pattern and, in some cases, confirmed with authentic standards (Table 1): Pyroglutamic acid (**1**), Pteroyl-D-glutamic acid (**2**), 2-hydroxy- 5-methoxy benzoic acid (**3**) and 2-hydroxybenzoic acid β-d-glucoside (**4**).

Only pteroyl-D-glutamic acid was not confirmed by MS/MS analysis due to the low intensity of the fragments generated. However, the presence of pyroglutamic acid, the only metabolite confirmed with an authentic standard, in the processing samples supports the occurrence of compound **2** in fresh strawberries. We initially hypothesized that compound **3** with *m*/*z* 167.0339 and a fragmentation pattern corresponding to a hydroxy-methoxy benzoic acid could be vanillic acid (4-hydroxy-3-methoxy benzoic acid), a metabolite naturally found in fruits such as banana, mango, blueberry, blackberry and strawberry and in processed products such as juice, wine, beer and cider [18]. However, its retention time did not match that of an authentic standard. Among the other isomers, considering its fragmentation pattern, this compound was tentatively identified as 2-hydroxy- 5-methoxy benzoic acid (5-methoxysalicylic acid).

**Figure 2:**
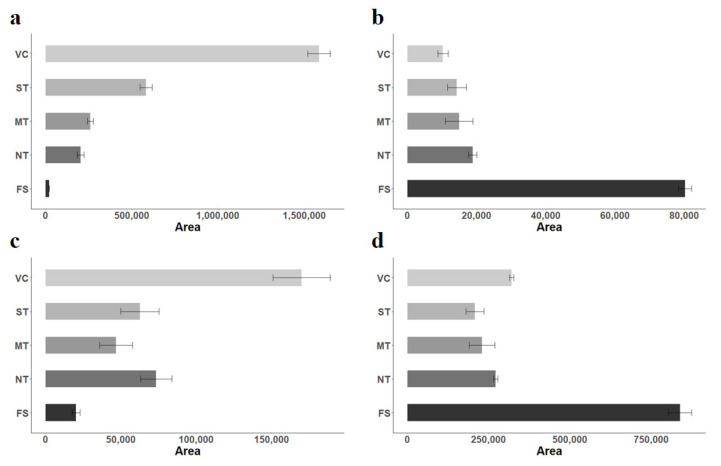
Bar plot of the metabolites identified in strawberry puree across the processing sample groups. X-axis: total abundance of the metabolite; Y-axis: treatment. (**a**) Pyroglutamic acid; (**b**) Pteroyl-D-glutamic acid; (**c**) 4-hydroxy-3-methoxy benzoic acid; (**d**) 2-hydroxybenzoic acid β-d-glucoside. Strawberry samples: Fresh strawberries (FS), No heat treatment (NT), mild heat treatment (MT), standard thermal treatment, vacuum concentration (VC).

The area of these potential markers was compared among the different processing treatments (Figure 2). Pyroglutamic acid (Figure 2a) showed an increasing trend mainly correlated with thermal processing. It was practically absent in the fresh fruits, and its presence was higher when increasing the temperature (thermal treatment) in the processing (FS < NT < MT < ST < VC). On the contrary, pteroyl-D-glutamic acid (Figure 2b) showed the opposite trend, a decrease in the area correlated with the thermal treatment applied to the samples. 2-Hydroxy- 5-methoxy benzoic acid (5-Methoxysalicylic acid) (Figure 2c) showed a similar trend to pyroglutamic acid, increasing with the processing intensity. Still, in this case, the temperature and cold-crushing (NT) could affect. 2-Hydroxybenzoic acid β-d-glucoside (Figure 2d) was a downregulated marker showing a decreasing trend correlated with the thermal processing intensity. However, other processing parameters applied during vacuum concentration (VC) apart from temperature could affect it.

Apple. After applying the pre-configured process layer to the apple products (see above), a total of 1723 entities were found in negative mode. According to the highest VIP value, *p*-value, and accuracy of the tentative database identification, a final list of 767 candidates was obtained. Finally, three entities were identified in databases using their accurate mass and MS/MS fragmentation pattern, and they were proposed as processing markers: di hydroxycinnamic acid glucuronide (**5**), caffeic acid (**6**), and lysoPE(18:3(9Z,12Z,15Z)/0:0) (**7**) (Table 1). Caffeic acid was also confirmed with an authentic standard. Compound **5** showed an MS/MS profile that matched the predicted spectrum of a hydroxycinnamic acid glucuronide.

**Figure 3:**
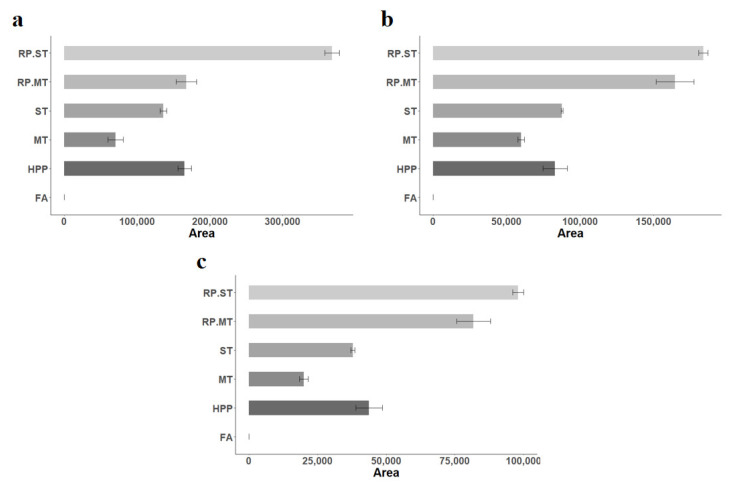
Bar plot of the metabolites identified in apple puree across the processing sample groups. X-axis: total abundance of the metabolite; Y-axis: treatment; (**a**) hydroxycinnamic acid glucuronide; (**b**) Caffeic acid; (**c**) LysoPE(18:3(9Z,12Z,15Z)/0:0). Apple samples: Fresh apple (FA), High-pressure processing (HPP), mild heat treatment (MT), standard thermal treatment (ST), reprocessed mild heat treatment (RP.MT), reprocessed standard thermal treatment (RP.ST).

After a first look, it seemed that it could be caffeic acid glucuronide. Still, its experimental fragmentation from 5 to 40 V produced the fragment at *m*/*z* 135 with low intensity, while in the theoretical MS/MS spectra of caffeic acid glucuronide, this was the major fragment. Accordingly, based on this evidence, it was impossible to specify the dihydroxycinnamic acid type or the glucuronide residue’s position. When the trend of these markers in the different processed samples was evaluated, it was observed that all of them (di-hydroxycinnamic acid glucuronide, caffeic acid, and lysoPE(18:3(9Z,12Z,15Z)/0:0)) were not detected in FA samples and were upregulated after processing. They showed an increasing trend correlated with processing, mainly with the thermal treatment applied to the samples (FA < MT < ST < RP.MT < RP.ST) (Figure 3). An exception was observed with the HPP treatment, which showed higher intensity than MT and was similar to ST but was subjected to lower thermal treatment. This behavior could be due to the high pressure applied during treatment, which could also affect these compounds’ presence.

## 3. Discussion

In the present study, untargeted metabolomics was successfully applied to identify changes in the metabolic profiles of strawberry and apple purees subjected to different thermal and non-thermal processing techniques [17]. Notably, multivariate analyses (PCA and PLS-DA) evidenced a clear separation between the other fruits’ treatments, highlighting a temperature-dependent effect. It was possible to discriminate each treatment based on its metabolomic profiling. It became evident that the presence or absence of temperature used during the crushing process is crucial in the change of the metabolomic profile of the final product. Thus, in the case of strawberries and apples, purees produced with cold crushing clustered together, while those extracted with heat treatment also formed a separate cluster. The effect of thermal processing on the overall metabolome of different foods has been previously reported using untargeted metabolomics approaches [10]. Previous studies have reported changes in the composition of peanuts due to dry roasting [19], in tiger nut milk caused by ultra-high temperature [20], and in black raspberry powder thermally treated at 95 °C [13]. In another study, thermal treatment influenced the final metabolite composition of vegetable purees differently depending on the blending and heating conditions applied [4]. Different molecules were identified as variables to discriminate between processed and fresh samples. Metabolites that increased or decreased along processing treatment, mainly affected by the temperature, were found.

**Figure 4:**
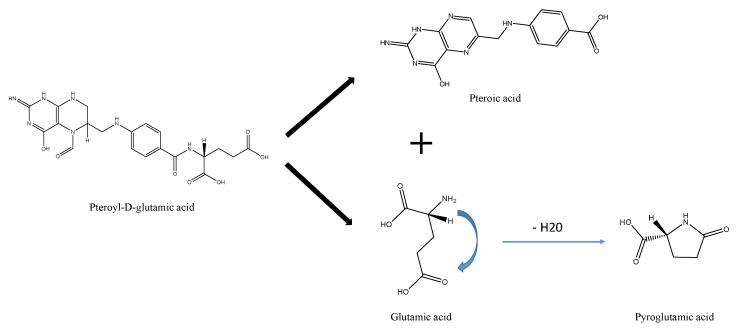
Diagram of pyroglutamic acid production.

In the case of strawberries, we found that pteroyl-D-glutamic acid could be a marker of non-processed fruits. Pteroyl-D-glutamic acid, the active metabolite of folic acid [21], is essential to prevent congenital malformations such as neural tube defects [22]. Folic acid and its derivatives are present in meat products (chicken liver), leafy green vegetables (spinach, parsley), grains (green beans, peas, chickpeas), and fruits (apple, orange, strawberry), with concentrations ranging from 1 to 580 µg of folate/100 g fresh weight [23,24]. Due to processing, a significant decrease in pteroyl-D-glutamic acid was observed in strawberries. These folates have been reported to be sensitive to oxygen exposure, light intensity, and thermal treatments [23]. Depending on the intensity of the processing conditions, folates could be transformed into their less available derivatives, resulting in losses ranging from 10% to 80% [25]. The decrease in pteroyl D-glutamic acid came with an increase in pyroglutamic acid, which was identified as an upregulated marker of processing in strawberry puree (Figure 2a). These results suggest that the pteroyl-D-glutamic acid in fresh strawberries is degraded with the temperature, releasing pteroic acid and glutamic acid. Then, glutamic acid is converted into pyroglutamic acid due to the loss of a water molecule and internal cyclization (Figure 4). Both metabolites, pteroic acid and glutamic, were not detected in samples, most likely due to the further degradation of pteroic acid into other molecules and the total conversion of glutamic acid to pyroglutamic acid. Transformation of glutamic acid or glutamine to pyroglutamic acid has been previously reported through enzymatic or non-enzymatic reactions [26,27]. Pyroglutamic acid formation after enzymatic reactions have been registered in dairy, meat, and fermented products, whereas that produced after non-enzymatic reactions, has been described for tomato puree and beer [28,29,30]. Prior research has also found that levels of pyroglutamic acid in foods influence various sensory attributes, such as metallic aroma and bitter taste [29,31]. In addition, evidence shows that L-pyroglutamic acid induces the formation of lower-molecular-weight colored products through non-enzymatic browning reactions [32]. Accordingly, accurately identifying this compound with the authentic standard supports pyroglutamic acid as a good marker of strawberry processing.

Conversely, 2-hydroxy-5-methoxy benzoic acid increased its intensity with thermal processing. It was identified as an upregulated marker of processing in strawberry purees according to the relevance of the thermal treatment, except for NT treatment containing the cold treatment exclusively, which produced an increase. Our results suggest that the thermal treatment may result in the release of 2-hydroxybenzoic (salicylic acid) from the β-d-glucoside conjugate. Then this compound could undergo oxidation and methylation reactions under thermal conditions. 2-Hydroxy-5-methoxy benzoic could also come from the methylation of 2,5-dihydroxybenzoic acid, but this is unlikely as we did not detect this metabolite in fresh strawberries. The 2-hydroxybenzoic acid β-d-glucoside (also known as glucosyl salicylate) was recognized as a downregulated processing marker according to the relevance of the thermal treatment in strawberry purees (Figure 2d), except for VC treatment where the vacuum applied increased its production specifically. Salicylic acid (SA) and its derivatives (usually called salicylates) are naturally present in plants, where they play an essential role in pathogen defense and the regulation of stress response [33]. Although there is no actual data about the presence of salicylic acid glucoside in fresh foods and vegetables, the content of salicylates in different food products has been reported in prior studies. For example, salicylic acid was present in free and conjugated forms in fruits (2–3140 µg/100 g dry weight) and vegetables (1–2693 µg/100 g dry weight), as well as in beverages, meat, dairy, and cereal products (2–1226 µg/100 g dry weight) [34]. Depending on the processing methods, different salicylate levels were observed between fresh and processed products [34]. Cooking particularly impacted the salicylate content, with vegetables boiled in water containing less salicylate than raw vegetables (beans, broccoli, cauliflower).

In the case of apple puree, caffeic acid, di-hydroxycinnamic acid glucuronide, and lysoPE(18:3(9Z,12Z,15Z)/0:0) were identified as upregulated markers of processing (Figure 3e–g) according to the relevance of the thermal treatment, except for the HPP treatment, which increased the metabolite profiles. The presence of caffeic acid, a hydroxycinnamic acid commonly found in fruits, vegetables, and processed products such as coffee, wine, and beer [32], was confirmed with an authentic standard. Caffeic acid, with numerous potential biological activities, is the main contributor to the diet among the hydroxycinnamic acids, with intakes ranging from 188 to 626 mg/day [32]. A negative correlation between the chlorogenic acid degradation and the progressive increments in caffeic acid, as a result of temperature, has been previously reported [33,34]. The increase in caffeic acid with processing can most likely be explained by two factors: the release from bound caffeic acid derivatives from plant structures due to the thermal processing and/or the thermal inactivation of polyphenol oxidase that degrades caffeic acid in fresh apples immediately after processing when thermal treatments are not applied.

Regarding di-hydroxycinnamic acid glucuronide, it has not been described in fresh products or as a result of any processing technique. However, other fruits have reported other glucuronides, such as kaempferol glucuronide and quercetin glucuronide [35]. In these cases, however, the glucuronides were naturally present in the fruits and not induced by processing (they are probably released better after thermal treatments). As mentioned earlier, thermal processing may release caffeic acid or other di-hydroxycinnamic acids from the tissue.

LysoPE(18:3(9Z,12Z,15Z)/0:0) (lysophosphatidylethanolamine) is a naturally present lipid with regulatory effects in senescence and ripening, found in the extraplastidial membranes of all plants and has been identified as a polar lipid in apple tissue and apple callus [36,37]. The temperature may act as an abiotic stress that triggers lipid-dependent signaling cascades [38]. Lysophospholipids are released from membrane phospholipids after the damage of the plant tissues (wounding, cutting, and probably also by non-thermal processing) through phospholipase activity. They are detected in the tissues immediately after cutting, as in fresh-cut lettuce tissues [39]. These lysophospholipids are then quickly metabolized by other enzymes of the jasmonate pathway (9-lipoxygenase, allene-oxide synthase, and other enzymes) to produce jasmonic acid and trigger phenolic compound biosynthesis to provide substrates for the development of tissue browning and tissue wound repairing [39]. In apple tissues, LysoPE can be released by cutting and other damages during processing and its enzymatic conversion to other metabolites of the jasmonic acid pathway can be prevented by the thermal treatments. This is why the LysoPE content correlates with the processing intensity as thermal processing can inactivate the jasmonate pathway enzymes and supports the occurrence of higher levels of lysophospholipids with thermal treatments.

The compounds identified in this study represent a good starting point as metabolites to be examined by future research. The variation in the metabolites’ intensity identified in this study is correlated with the degree of processing, in terms of thermal processing, except for the exclusively cold treatment, in the case of strawberry puree, and the pressure treatment, in the case of apple puree. This might contribute to detecting new processing markers, likely to help various stakeholders. In particular, the food industry could use them to optimize the degree of processing of food products. In contrast, academia or governments may use the markers to classify foods based on the degree of processing, which is currently misclassified following attributes not directly related to processing (e.g., NOVA classification system [40]). Moreover, such insights could be used as criteria to measure the degree of food naturalness, which is increasingly demanded by consumers [5,41].

## 4. Materials and Methods

### 4.1. Chemicals

Authentic standards of caffeic acid (purity > 96%), pyroglutamic acid (purity > 96%), vanillic acid (purity > 96%), salicylic acid (purity > 96%) and gentisic acid (purity > 96%) were purchased from Sigma-Aldrich (St. Louis, MO, USA). Methanol, acetic acid, acetonitrile, and water 0.1% (v/v) formic acid were from J.T. Baker (Deventer, The Netherlands), and formic acid was obtained from Panreac (Barcelona, Spain). Ultrapure water was obtained through the Milli-Q system (Millipore Corp., Bedford, MA, USA).

### 4.2. Processing of Strawberry and Apple Purees

**Figure 5:**
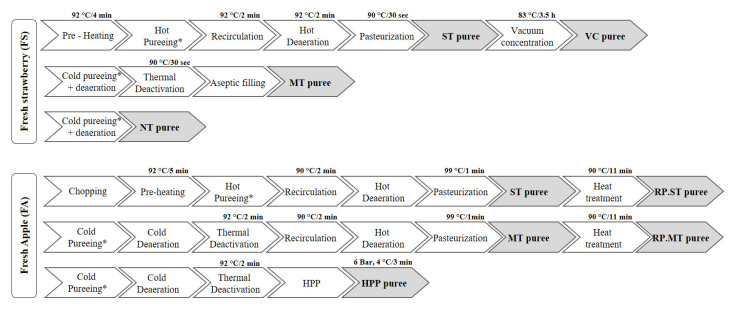
Diagram of the industrial processing techniques employed to obtain strawberry (Fresh strawberries (FS), No heat treatment (NT), mild heat treatment (MT), standard thermal treatment, vacuum concentration (VC)) and apple (Fresh apple (FA), High-pressure processing (HPP), mild heat treatment (MT), standard thermal treatment (ST), reprocessed mild heat treatment (RP.MT), reprocessed standard thermal treatment (RP.ST)) purees. * Removal of peel and seeds.

This research involves the study of two fruit products, strawberry, and apple purees, processed through different industrial processing techniques, as reported previously [17] (Figure 5). A total of 15 tons of strawberries (*Fragaria × ananassa*) of cultivar Primoris (Huelva, Spain) and 10 tons of apples (*Malus domestica*) of cultivar Golden Delicious (Zaragoza, Spain) were harvested ripe and transported under refrigeration conditions to the processing facility. The total amount of fruits was equally distributed to the different industrial processing technologies used to obtain purees from strawberries and apples [17].

In general, two extraction techniques were used to obtain the purees, cold crushing, and hot crushing. Cold crushing was performed in a cold extraction line, including deaeration and enzymatic deactivation. Hot crushing was performed in a processing line with a turbo extractor, hot deaerator, and pasteurizer. In the case of cold crushing, fruits, seeds, stems, and skin were separated from the mash after the crushing. For strawberries, the puree extracted by cold crushing was divided into two. One part was subjected to a mild heat treatment of 90 °C/30 s, whereas the other did not receive any additional heat treatment. However, for the puree extracted by hot crushing, entire strawberries were preheated (92 °C/4 min) and then crushed. The hot mash was deaerated (92 °C/2 min) and pasteurized at 90 °C for 30 s resulting in a standard treated puree. Finally, to produce vacuum-concentrated puree, ST puree was subjected to a vacuum concentration (0.3–0.4 Bar) at 83 °C for 3.5 h. In the case of apples, the puree obtained after the cold crushing was thermally deactivated (92 °C/2 min) and divided into two portions. One part was packed and treated by high-pressure processing (6 bar, 4 °C/ 3 min), whereas the other part was hot deaerated and pasteurized at 99 °C for 1 min, obtaining mildly treated puree. To obtain the purée by standard thermal treatment, apples were chopped, pre-heated at 92 °C for 5 min, and hot crushed, to finally being refined, separating the skin and seeds from the puree. The obtained puree was deaerated and pasteurized at 99 °C for 1 min. To evaluate the effects of re-processing, samples of mild heat-treated puree and standard thermal-treated apple puree stored at 24 °C for six months were subjected to an additional thermal treatment of 90 °C for 11 min re-processed apple purees. In both studies, fresh fruits were used as a control. After the different processing, samples of strawberry and apple purees were taken. Their control samples were lyophilized to remove the moisture and ground into powder using a dry bean blender to homogenize the sample.

### 4.3. Sample Preparation

Extractions for strawberry and apple samples were carried out as previously described by Buendía et al., 2010 [42] and Jakobek et al., 2013 [43], respectively, with some modifications focused on the extraction of as many compounds as possible for the untargeted analysis. A total of 50 mg of lyophilized samples was extracted with 1 mL of methanol/water/acetic acid (70:29:1, *v*/*v*/*v*) for strawberries and methanol/water (70:30, *v*/*v*) for apples. The samples were homogenized in a vortex for one minute and then sonicated for 30 min at room temperature. Subsequently, these were centrifuged for 15 min at 20,627× *g* at 12 °C. The resultant supernatant was filtered through a 0.22 µm PVDF filter before UPLC-MS analysis. Three replicates for each condition were extracted and analyzed.

### 4.4. UPLC-ESI-QTOF-MS Analysis

Samples were analyzed using an Agilent 1290 Infinity LC system coupled to the 6550 Accurate-Mass Quadrupole time-of-flight (QTOF) (Agilent Technologies, Waldbronn, Germany) using an electrospray interface (Jet Stream Technology). Chromatographic separation was carried out on reversed-phase C18 column (3.0 × 100 mm, 2.1 µm particle size) (ACE Excel, Scotland) at 30 °C, using as mobile phases water + 0.1% formic acid (Phase A) and acetonitrile + 0.1% formic acid (Phase B) with a flow rate of 0.5 mL/min. The following gradient was used: 0–7 min, 5–18% B; 7–17 min, 18–28% B; 17–22 min, 28–50% B, 22–27 min, 50–90% B, 27–29 min, whereafter the gradient comes back to the initial conditions (5% B), which are maintained for 6 min. The injection volume was 5 µL. The optimal conditions of the electrospray interface were as follows: gas temperature 280 °C, drying gas 11 L/min, nebulizer 45 psi, sheath gas temperature 400 °C, and sheath gas flow 12 L/min. Spectra were acquired in the *m*/*z* range 100–1100 in negative and positive mode, and fragmentor voltage was 100 V. MS/MS product ion spectra were collected at an *m*/*z* range of 50–1000 using a retention time window of 1 min, collision energy of 10 and 20 eV and an acquisition rate of 4 spectra/s.

### 4.5. Untargeted Metabolomics Data Treatment

The data generated by UPLC-ESI-QTOF-MS metabolomics system were acquired in profile mode. The raw data were exported to Profinder software (Agilent Technologies) for pre-processing procedures and to build the data matrix for further processing and data treatment. Independent data matrixes were created to process and analyze strawberry and apple samples separately for each negative and positive polarity. The data matrixes were imported in parallel to the Metaboanalyst online platform and Mass Profiler Professional (MPP, Agilent Technologies). Data processing was performed before univariate and multivariate analysis, including data log transformation and Pareto scaling [44].

Regarding multivariate analysis, the principal component analysis (PCA) and partial least squares discriminant analysis (PLS-DA) models of the final data matrix were created using the Metaboanalyst platform to describe the total variance of the full data set and figure out the discriminations groups under data matrix criteria. The VIP (variable importance in projection) score value (VIP > 1) obtained by the discriminant analysis was used for candidate selection. After the multivariate analysis evaluation, univariate operations were performed in MPP software. Data treatment through MPP software included filters by frequency of the data matrix to reduce the sample variability within each study group and the ANOVA statistics analysis (corrected p-value cut-off: 0.05; p-value computation: Asymptotic; Multiple Testing Correction: Benjamini–Hochberg). The candidates must be significant, at least between the extreme samples group (FS and VC in the case of strawberry; FA and RP.MT or RP.ST in the case of apple puree). The VIP > 1 score and p-value were used to create the candidate list for evaluating the processing treatment.

After the selection of the candidates, the authentic standards and the MS/MS spectra data of those ions were used for metabolite confirmation. Metlin and MassBank of Noth America (MoNA) databases were used for checking the tentative identification. In addition to the databases, the competitive fragmentation modeling for metabolite identification (CFM-ID) software was complementary to confirm the metabolites. The positive polarity confirmed no metabolites by MS/MS spectra fragmentation or authentic standards. This may be due to the limitation of the method for achieving good ionization results and the unavailability of authentic standards. Despite this, the data acquired in positive polarity were also uploaded to the data repository, and the experimental data in positive polarity could be used in future investigations. The metabolomics data were deposited in the Metabolights database (https://www.ebi.ac.uk/metabolights/reviewerf89c976a-d48f-4218-9e57-64aa1cce52dd) (accessed on 5 May 2022).

## 5. Conclusions

Processing is a relevant and pervasive practice in the food industry that can affect the composition and quality of foods. Untargeted metabolomics has been demonstrated to be a valuable tool to visualize changes in the metabolic profile of strawberry and apple purees subjected to different processing techniques in a real industry case. The study supposes a promising source of candidates to be confirmed in further investigation and applied in new treatments of the food industry in the future.

Several metabolites showed changes according to the relevance of thermal conditions but also according to the exclusively cold treatment, in the case of strawberry puree, and the pressure treatment, in the case of apple puree. These findings suggest the possibility of studying the isolated impact of these variables. Seven of these compounds were identified and were proposed as potentially powerful markers to evaluate the processing degree of strawberry and apple puree products. The pyroglutamic acid, pteroyl-d-glutamic acid, 2-hydroxy-5-methoxybenzoic acid, and 2-hydroxybenzoic acid β-d-glucoside were identified in strawberry and di-hydroxycinnamic acid glucuronide, caffeic acid and lysoPE(18:3(9Z,12Z,15Z)/0:0) in apple purees. The metabolites confirmed as pyroglutamic acid in the case of strawberry puree and caffeic acid in the apple puree are potential candidates to be validated in a specific protocol by the food industry due to the availability of authentic standards. This study opens a new field for applying untargeted metabolomics to find markers of processing produced in the food industry.

## Figures and Tables

**Table 1:** Metabolites identified and confirmed by MS/MS in strawberry and apple samples.

ID	m/z	Name	Formula	RT	Polarity	Regulation	MS/MS Fragments
**1**	128.0344	Pyroglutamic acid *	C_5_H_7_NO_3_	1.37	NEG	UP	128.0338; 85.0287; 82.0285; 72.0091
**2**	472.1577	Pteroyl-D-glutamic acid ^a^	C_20_H_23_N_7_O_7_	3.70	NEG	DOWN	Unclear fragments
**3**	167.0339	2-hydroxy-5-methoxy benzoic acid	C_8_H_8_O_4_	7.52	NEG	UP	108.0217;109.0243;152.0109;123.0019; 167.0360
**4**	299.0766	2-hydroxybenzoic acid beta-d-glucoside	C_13_H_16_O_8_	2.16	NEG	DOWN	137.0246; 179.0437; 299.0761
**5**	355.0666	Dihydroxycinnamic acid glucuronide	C_15_H_16_O_10_	2.88	NEG	UP	207.0297; 265.0358; 247.0250;193.0609;191.0555; 135.0488
**6**	179.0345	Caffeic acid *	C_9_H_8_O_4_	5.78	NEG	UP	135.0455; 134.0369
**7**	474.2621	LysoPE(18:3(9Z,12Z,15Z)/0:0)	C_23_H_42_NO_7_P	25.01	NEG	UP	474.2626; 277.2177; 214.0487; 152.9955

^a^ Tentative identification; * Confirmed by an authentic standard; ID 1-4: detected in strawberry; ID 5-7: detected in apple. MS/MS fragments compared with Metlin database, MassBank of North America (MoNA), and calculated by CFM-ID spectrum prediction.

## Data Availability

The raw metabolomics data was deposited in the Metabolights database (https://www.ebi.ac.uk/metabolights/reviewerf89c976a-d48f-4218-9e57-64aa1cce52dd) (accessed on 5 May 2022).
